# Depth of colorectal-wall invasion and lymph-node involvement as major outcome factors influencing surgical strategy in patients with advanced and recurrent ovarian cancer with diffuse peritoneal metastases

**DOI:** 10.1186/1477-7819-11-64

**Published:** 2013-03-09

**Authors:** Angelo Di Giorgio, Maurizio Cardi, Daniele Biacchi, Simone Sibio, Fabio Accarpio, Antonio Ciardi, Tommaso Cornali, Marialuisa Framarino, Paolo Sammartino

**Affiliations:** 1 Department of Surgery ‘Pietro Valdoni’, Sapienza University of Rome Azienda Policlinico Umberto I, viale del Policlinico, 155, Rome, 00161Italy; 2 Department of Experimental Medicine, Sapienza University of Rome - Azienda Policlinico Umberto I, viale del Policlinico, 155, Rome, 00161, Italy; 3 Department of Obstetrics and Gynecology, Sapienza University of Rome Azienda Policlinico Umberto I, viale del Policlinico, 155, Rome, 00161, Italy

**Keywords:** Ovarian cancer, Peritoneal metastases, Colorectal resection, Depth of bowel wall invasion, Mesenteric lymph node involvement

## Abstract

**Background:**

More information is needed on the anatomopathological outcome variables indicating the appropriate surgical strategy for the colorectal resections often needed during cytoreduction for ovarian cancer.

**Methods:**

From a phase-II study cohort including 70 patients with primary advanced or recurrent ovarian cancer with diffuse peritoneal metastases treated from November 2000 to April 2009, we selected for this study the 52 consecutive patients who needed colorectal resection. Data collected included type of colorectal resection, peritoneal cancer index (PCI), histopathology (depth of bowel-wall invasion and lymph-node spread), cytoreduction rate and outcome. Correlations were tested between possible prognostic factors and Kaplan-Meier five-year overall and disease-free survival. A Cox multivariate regression model was used to identify independent variables associated with outcome.

**Results:**

In the 52 patients, the optimal cytoreduction rate was 86.5% (CC0/1). In all patients, implants infiltrated deeply into the bowel wall, in 75% of the cases up to the muscular and mucosal layer. Lymph-node metastases were detected in 50% of the cases; mesenteric nodes were involved in 42.3%. Most patients (52%) had an uneventful postoperative course. Operative mortality was 3.8%. The five-year survival rate was 49.9% and five-year disease-free survival was 36.7%. Cox regression analysis identified as the main prognostic factors completeness of cytoreduction and depth of bowel wall invasion.

**Conclusions:**

Our findings suggest that the major independent prognostic factors in patients with advanced ovarian cancer needing colorectal resections are completeness of cytoreduction and depth of bowel wall invasion. Surgical management and pathological assessment should be aware of and deal with dual locoregional and mesenteric lymphatic spread.

## Background

Epithelial ovarian cancer is the fifth most frequent cause of cancer death in women and remains the leading cause of gynecologic cancer-related deaths in the US and Europe [1,2]. Although aggressive surgical cytoreduction and platinum- plus taxane-based chemotherapy have in recent years improved median overall survival in advanced ovarian cancer [3,4], relapse rates reach 80% and long-term cure rates languish between 20 and 30% [4-6]. After a meta-analysis demonstrated the prognostic value of maximal cytoreduction [7], the definition of desirable surgical outcome switched from so-called ‘optimal debulking’ with variable residual disease up to 1 to 2 cm [8], to microscopic residual disease alone [9-12], justifying more aggressive surgery. To improve the outcome in treating patients with primary advanced or recurrent ovarian cancer, given the known benefits of normothermic intraperitoneal chemotherapy [13], some have combined cytoreduction (peritonectomy procedures) with hyperthermic intraperitoneal chemotherapy (HIPEC) [14-16].

As Hoffman *et al*. report, in about 26% of these patients the various visceral resections needed to reach maximal cytoreduction include colorectal resections [17]. Besides wide consensus on the need for colorectal resections to reduce residual disease, many investigators also emphasize the acceptable complications rate and the fact that, in most cases, the colorectum can be resected, avoiding an intestinal stoma [18,19]. Some compared the long-term results in patients who needed a colorectal resection (for example a rectosigmoid resection) and those in whom it could be avoided simply by removing the peritoneum in the Douglas pouch. Hardly surprisingly, they found that the results in the two groups overlapped [20] because outcomes in patients with advanced ovarian cancer depend crucially on the completeness of cytoreduction achieved, regardless of the surgical procedures used. No studies have yet clearly identified prognostic factors linking colorectal resection required during cytoreduction in these patients with survival.

Seeking a better guide to surgical management, we designed this study to assess anatomopathological outcome variables correlated with large-bowel involvement in patients with primary advanced or recurrent ovarian cancer with diffuse peritoneal metastases. To do so, from a larger consecutive series of prospectively enrolled patients with advanced ovarian cancer treated in our center with peritonectomy procedures and HIPEC, we selected for study those patients in whom cytoreduction required colorectal resections. Univariate and multivariate regression analyses were used to determine the prognostic value of several outcome variables including completeness of cytoreduction, depth of bowel wall invasion and lymph node spread.

## Methods

From a phase II trial including a cohort of 70 patients with primary advanced or recurrent ovarian cancer with diffuse peritoneal metastases treated with peritonectomy and HIPEC from November 2000 to April 2009, we selected for study the 52 consecutive patients who had ovarian implants invading at least a single colorectal segment confirmed by intraoperative assessment and requiring colorectal resection. Colorectal resection was required because macroscopic disease in the colorectal segment, pouch or mesentery or both made it impossible to clear *in situ* malignant implants or attempt serosal stripping of the visceral surface. The inclusion criteria for peritonectomy were age younger than 75 years; histologically or cytologically confirmed diagnosis; performance status 0 to 2 (WHO); adequate cardiac, renal, hepatic and bone marrow function; resectable disease; and informed written consent. The exclusion criteria were extra-abdominal metastases; other malignancies except breast cancer; unresectable disease; active infection or severe associated medical conditions. The extent of malignant peritoneal disease was assessed with the peritoneal cancer index (PCI) - a scoring system ranging from 0 to 39 according to the extent of metastasis into the peritoneal cavity [21]. The completeness of cytoreduction (CC) was scored as CC0: no residual disease; CC1: residual nodules measuring less than 2.5 mm; CC2: residual nodes measuring between 2.5 mm and 2.5 cm; and CC3 residual nodules greater than 2.5 cm [22]. Aggressive surgical cytoreduction followed the standard accepted techniques for peritonectomy [23].

When the tumor mass involved the pelvis, cul-de-sac, uterus and adnexa and rectosigmoid colon, or recurrent bulky disease involved the pelvis, maximal cytoreductive surgery usually comprised an *en bloc* resection including the internal female genitalia or pelvic recurrence along with the rectum and sigmoid, according to the pelvic peritonectomy technique [23]. Our policy envisaged a low distal rectal section just above the pelvic diaphragm, comprising the mesorectum, leaving a rectal stump no longer than 5 cm. The inferior mesenteric artery was tied at its origin from the aorta and the vein at the ligament of Treitz. Patients with peritoneal implants involving the cecum, appendix, terminal ileum or ascending colon underwent a standard right hemicolectomy. In patients with diffuse colonic involvement, every effort was made to preserve as much colon as possible by clearing malignant implants from the surface [24]. Patients with peritoneal spread involving the pelvis and all colonic segments, with nodules penetrating deeply into the colonic wall, underwent total colectomy and rectal resection.

When each surgical procedure ended, HIPEC was given under general anesthesia with the closed technique and during hemodynamic monitoring. Cisplatin at a dose of 75 mg/m^2^ at inflow temperatures ranging from 42 to 43°C was given for 60 minutes. The technique used for HIPEC has been detailed in our preceding work [14].

The patients were admitted to the intensive care unit (ICU) for at least the first 24 hours after operation. Cisplatin toxicity, surgical complications and adverse events were graded from 0 to V according to the National Cancer Institutes Common Toxicity Criteria [25,26].

All patients were followed up by members of the surgical staff and referred to the medical oncologic staff to plan systemic chemotherapy. Outpatient clinic visits for follow-up assessment were scheduled according to the individual patient’s conditions.

Anatomopathological features studied in detail for each patient included the depth of malignant bowel wall invasion (serosal, muscle and mucosal layers) and lymph node spread. Lymph nodes were identified, taking into account dual polarity (locoregional and mesenteric) lymph node spread, and classified by site, distinguishing between locoregional ovarian (pelvic, obturator and interaortocaval stations) and mesenteric (pericolic, and mesorectal) nodes.

A multiple regression analysis was used to test correlations between possible prognostic factors. Survival was expressed as median months, and as five-year overall and disease-free rates. The Kaplan-Meier method was used to construct survival curves and the log-rank test (univariate analysis) was used to assess the significance of differences between curves. The Cox multivariate regression model was used to determine the prognostic value of independent variables. *P* values <0.05 were considered to indicate statistical significance. The NCSS package (NCSS, LLC, Kaysville, UT, USA) was used to analyze the database and conduct statistical tests.

All patients gave their informed consent to the study. The procedures were approved by the institutional review board at Policlinico Umberto I Rome and the research complied with the Helsinki Declaration.

## Results

Patients’ demographic and clinical characteristics are reported in Table 1.

**Table 1:** Patients’ demographic and clinical characteristics (52 patients)

**Characteristics**	**N (%)**
Age (years)	mean 62 (range 32–76)
Primary cytoreduction	30 (57.7%)
Secondary cytoreduction	22 (42.3%)
Previous chemotherapy	
None	22 (42.3%)
Adjuvant	22 (42.3%)
Neoadjuvant	8 (15.4%)
Performance status (WHO)	
0	18 (34.6%)
1	17 (32.7%)
2	17 (32.7%)
Intestinal obstruction	
Absent	35 (67.3%)
Present	17 (32.7%)
Ascites	
Absent	19 (36.5%)
Present	33 (63.5%)
Comorbidity	
Absent	38 (73.1%)
Present	14 (26.9%)
Ca125 level	mean 579.25 U/ml (range 15 to 6800 U/ml)
Peritoneal cancer index (PCI)	mean 18.8 (range 6 to 28)

In the 52 patients selected for study because malignant disease required colorectal resections, peritonectomy procedures during cytoreduction comprised a mean number of 7.5 resections per patient (Table 2). During primary cytoreductive surgery, ovarian locoregional lymph nodes were routinely dissected whereas during secondary cytoreductive surgery, they were dissected in only 10 of the 22 patients who underwent cytoreduction because the procedure had not been done at the first operation. All the 52 patients selected because they underwent colorectal resection had site-specific mesenteric lymphadenectomy (Table 2).

**Table 2:** Types of resection during peritonectomy (52 patients)

**Type of colorectal resection**	**N (%)**
Rectal resection + left hemicolectomy	24 (46.1%)
Rectal resection + total colectomy	18 (34.6%)
Rectal resection + right hemicolectomy and left hemicolectomy	6 (11.5%)
Right hemicolectomy	4 (7.7%)
Total	52 (100%)
Associated visceral resections	N (%)
Hysterectomy ± adnexectomy	35 (67.3%)
Pelvic mass resection	6 (11.5%)
Omental resection	49 (94.2%)
Liver resection	2 (3.8%)
Cholecystectomy	14 (26.9%)
Splenectomy	28 (53.8%)
Small bowel resection	16 (30.8%)
Appendectomy	11 (21.1%)
Total cystectomy	1 (1.9%)
Bladder resection	3 (5.8%)
Total peritonectomy	10 (19.3%)
Partial peritonectomy	42 (80.7%)
Abdominal wall resection	13 (25%)
Resection or reduction of cancer implants	51 (92.7%)
Locoregional + mesenteric lymphadenectomy	40 (76.9%)
Mesenteric lymphadenectomy alone	12 (23.1%)
Other types of resection (pancreatic, gastric, vaginal resection)	4 (7.7%)
Total	337 (100%)
Total surgical procedures	389 (mean 7.5)

Complete cytoreduction (CC-0) was achieved in 28 (53.8%) of the patients, CC-1 in 17 (32.7%), CC-2 in 5 (9.6%) and CC-3 in 2 (3.8%), yielding an 86.5% rate of optimal cytoreduction (CC-0 and CC-1).

Peritonectomy procedures lasted a mean 510 minutes (range 300 to 780) including 60 minutes HIPEC. All operations led to major blood loss (mean 1700 ml, range 500 to 4900) and required intraoperative blood (mean 4, range 2 to 8 units) and plasma (mean 6, range 2 to 10 units) transfusions.

Most patients (52%) had an uneventful postoperative course. The only HIPEC-related adverse events were renal cisplatin toxicity (2 cases, grade 1 in one and grade 2 in the other patient), and medical treatment reversed both drug-induced reactions.

Grade I/II complications developed in 23.1%, grade III in 7.7% and grade IV in 13.4% of the patients. Of the seven patients with grade IV complications, six underwent a second operation, two for colonic fistulas both unrelated to colorectal anastomoses but caused by the surgical maneuvers needed to ablate colonic implants, two for postoperative bleeding, one patient for a small bowel fistula due to a perforation that developed during pancreatitis, and one for an abdominal eventration. One patient returned to the ICU on postoperative day 6 after a myocardial infarction. Two patients died of pulmonary embolism despite anticoagulant treatment (operative mortality 3.8%). Mean postoperative stay was 21.6 days (range 8 to 90). After hospital discharge all but two patients were fit for postoperative systemic chemotherapy.

Histopathology typed most tumors (71.5%) as serous carcinomas followed by mucinous carcinoma (17.3%), and endometrioid cancer (11.5%). In all 52 cases malignant implants infiltrated the colorectal wall: in 13 (25%) the serosa; in 35 (67.3%) the muscular; and in 4 (7.7%) reaching the mucosal layer. Multiple regression analysis showed that the depth of bowel wall involvement significantly correlates with more extensive peritoneal spread (PCI >18) and with overall lymph node metastases (Table 3).

**Table 3:** Anatomopathological outcome variables (52 patients)

**Depth of colorectal wall involvement (52 patients)**		**N (%)**	
Serosal layer		13 (25%)	
Muscular layer		35 (67.3%)	
Mucosal layer		4 (7.7%)	
Multiple regression report			
Dependent variable	Independent variable	T value	Prob level
Depth of colorectal wall involvement	n colorectal resections >1	−0.274	n.s.
	total colectomy	0.341	n.s.
	PCI >18	3.709	0.0005
	Lymph node metastases	2.990	0.004
**Lymph node site and status (52 patients)**		**N (%)**	
N0		26 (50%)	
N+ locoregional lymph nodes (A)		4 (7.7%)	
N+ mesenteric lymph nodes (B)		16 (30.8%)	
A + B		6 (11.5%)	
**Mesenteric lymph node status and possible predictive factors (52 patients)**		**Mesenteric nodes**	
		node positive	node negative
		n° patients	n° patients
Depth of colorectal wall involvement (*P* = ns)			
Serosal layer		6	7
Muscular and mucosal layers		16	23
Number of colorectal resections (*P* <0.01)			
Single resection		7	21
Double resection		5	1
Total colectomy		10	8
Locoregional lymph node status (*P* = ns)			
Positive		6	4
Negative		16	26
Peritoneal cancer index (*P* = ns)			
PCI < 18		7	15
PCI ≥ 18		15	15

Seeking dual polarity nodal spread, in the 52 patients we examined a mean of 24.3 (range 7 to 49) locoregional ovarian lymph nodes and a mean of 27.3 (range 14 to 60) mesenteric and mesorectal nodes per patient. Lymph node metastases were detected in 50% of the patients and involved the mesenteric nodes more frequently than the ovarian locoregional nodes (30.8% vs. 7.7%), and in 11.5% of the cases involved both stations (Table 3). Of the 48 patients who underwent a rectal resection, 20 (41.6%) had mesorectal lymph node metastases. Of the prognostic variables assessed (depth of colorectal wall invasion, number of colorectal resections, presence of locoregional node metastases and extent of peritoneal involvement), the only predictive factor that correlated significantly with mesenteric node metastases was the number of colorectal resections (*P* <0.01) (Table 3).

At a mean follow-up of 73.5 months (range 36 to 118), the overall five-year Kaplan-Meier survival rate in the 52 patients was 49.9% and the disease-free survival rate was 36.7%. The overall median survival was 28 months and the median disease-free survival was 20 months. For the patients optimally debulked (CC-0 and CC-1) survival reached a value of 35.5 months overall and 32.5 months disease-free.

Univariate analysis (log-rank test) identified as the prognostic factors significantly correlated with long-term (five-year) survival, the CC score (*P* <0.002) and degree of colorectal-wall involvement (*P* <0.037). Cox regression model verifying the relationship between survival and combined prognostic factors confirmed that the only independent variables significantly influencing patients’ survival were CC score (*P* <0.003) and depth of colorectal-wall involvement (*P* <0.004) (Table 4).

**Table 4:** Prognostic factors and five-year survival by univariate and multivariate analysis (52 patients)

**Prognostic factors**	**Variables**	**Five-year survival**	P* (univariate analysis)	P** (multivariate analysis)
Patients’ age (years)	<62	52.2%	0.49	0.79
	≥62	41.2%		
Ascites	Absent	39.2%	0.97	0.08
	Present	47.5%		
Obstruction	Absent	56.7%	0.64	0.59
	Present	25.4%		
Cytoreduction	Primary	47.2%	0.98	0.73
	Secondary	37.4%		
Peritoneal cancer index	<18	53.5%	0.08	0.17
	≥18	33.6%		
Completeness of cytoreduction score	CC-0	55%	0.002	0.003
	CC 1-3	25.7%		
Depth of colorectal wall involvement	Serosal layer	72.7%	0.037	0.004
	Muscular layer	33.1%		
	Mucosa layer	0%		
Lymph node status	N0	41.3%	0.67	0.76
	N+ (A)	75%		
	N+ (B)	42.5%		
	N+ (A + B)	27.7%		

P* by log-rank test; P** by Cox regression model. A, locoregional lymph nodes; B, mesenteric lymph nodes.

## Discussion

For this study, from a larger series of patients who underwent cytoreductive surgery plus HIPEC for primary advanced or recurrent ovarian cancer with diffuse peritoneal metastases, we explicitly selected those in whom cytoreduction included colorectal resections. This selection criterion allowed us to confirm and extend current knowledge on anatomopathological factors correlated with large bowel involvement that could influence outcome and survival. In our series, surgical cytoreduction called frequently for colorectal resections (52 in the original series of 70 patients, 74.3%) because, as a tertiary referral center for the integrated treatment of peritoneal carcinomatosis, the patients we treat typically have a high index of peritoneal spread (mean PCI 18.8, range 6 to 28).

When we compared our results with those of others who analyzed the surgical specimens removed in detail [20,27-35] - investigating the depth of invasion into the large bowel wall, mesenteric lymph node spread, their relation to survival, and overall survival in patients who underwent optimal cytoreduction - several distinctive findings emerged (Table 5). The major observation was that the Cox regression analysis identified the depth of colorectal wall invasion as an independent prognostic factor statistically equal to the amount of residual disease (Table 4). None of the patients in whom large bowel wall invasion reached the mucosa survived more than three years. The considerable prognostic importance that the depth of invasion into the large bowel wall attained in our series partly agrees with the only two similar previous reports that underlined this finding [29,31]. In the series conducted by Scarabelli *et al*. the depth of large bowel wall invasion achieved prognostic value in the multivariate analysis only in patients with co-existing mesenteric lymph node metastases [29]. Conversely, in the patients studied by Park *et al.* this variable attained significant prognostic value in the log-rank test only for the disease-free interval whereas the significance disappeared when they analyzed overall survival [31]. We attribute the strong prognostic value for the depth of large bowel wall invasion to the lower percentage of patients with serosal invasion alone in our series than in others (serosal layers 25% vs. a mean of 44.5%) and to the higher mean percentage of patients with malignant disease infiltrating deep into the large bowel wall (muscular and mucosal layers 75% vs. mean 55.4%) (*P* <0.01) (Table 5). The worse outcome in patients with deeper large bowel wall involvement can be explained by the multiple regression analysis showing that the depth of bowel wall involvement correlates significantly with more extensive peritoneal spread (PCI >18) and with overall lymph-node metastases (Table 3).

**Table 5:** Colorectal involvement in advanced ovarian cancer: literature review

**Author/year**	**Patients (number)**	**Depth of bowel wall involvement (%)**	**Mesenteric nodal involvement (%)**	**Prognostic significance of pathological variables**	**Survival of optimally debulked patients**
*O’Hanlan*[27]*(1995)*	66	Serosa 41	72.7	Trend for patients with mesenteric nodal involvement to fail sooner	26 months
		Muscolaris 42			
		Submucosa 14			
		Mucosa 3			
*Lax*[28]*(1998)*	31	Serosa 45	64.5	Trend for patients with mesenteric nodal involvement to fail sooner	21 months
		Muscolaris 36			
		Mucosa 19			
*Scarabelli*[29]*(2000)*	66	Muscolaris 100	37.9	Prognostic significance of depth of bowel wall involvement at multivariate analysis for patients with mesenteric node metastases	5 years
					42.2 %
*Hertel*[30]*(2001)*	73	Serosa 38.5	Not reported	Not reported	Not reported
		Muscolaris 42.4			
		Mucosa 19.1			
*Park*[31]*(2006)*	46	Serosa 71.7	15.2	Prognostic significance of depth of bowel wall involvement only for disease-free survival	32 months
		Muscolaris + mucosa 28.3			
*Tebes*[32]*(2006)*	99	Serosa 33.3	Not reported	Not reported	Not reported
		Muscolaris 39.4			
		Submucosa 13.1			
		Mucosa 14.2			
*Salani*[33]*(2007)*	31	Serosa 48	93.5	Not reported	Not reported
		Muscolaris 29			
		Submucosa 7			
		Mucosa 16			
*Gallota*[20]*(2011)*	71	Serosa 45	51	NO	38 months overall
		Muscolaris 25.4			
		Submucosa 12.7			30 months disease-free
		Mucosa 16.9			
*Baiocchi*[34]*(2011)*	41	Serosa 34.1	70.7	Not reported	Not reported
		Muscolaris 31.7			
		Submucosa 14.6			
		Mucosa 19.5			
*Gouy*[35]*(2012)*	47	Serosa 61.7	40.4	NO	4 years 60 %
		Muscolaris 19.1			
		Submucosa 6.3			
		Mucosa 12.7			
*Present series*	52	Serosa 25	42.3	Prognostic significance of depth of bowel wall involvement at multivariate analysis	35.5 months overall
		Muscolaris 67.3			
		Mucosa 7.7			32.5 months disease-free

The second finding in our series, in which cytoreductive surgery with colorectal resection invariably included mesenteric lymphadenectomy, was the 42.3% frequency of metastatic spread to the mesenteric lymph nodes. This frequency accords with the figure reported by Dvorethsky *et al*. reported in an autopsy study on 100 patients [36]. In the studies we analyzed (Table 5), the wide variability in metastatic mesenteric node spread - from 15.2% reported by Park *et al.*[31] to 93.5% reported by Salani *et al.*[33] - presumably depends on the lack of a systematic surgical protocol for mesenteric lymphadenectomy in these patients [33]. Some investigators underline that the frequency of mesenteric lymph node spread correlates significantly with certain pathologic variables [28,31,33]. For example, Park *et al.* related malignant mesenteric lymph node spread to the depth of colorectal wall invasion [31], Salani *et al.* to retroperitoneal lymph-node spread [33] and Lax *et al.* to the amount of colorectum resected [28]. In their later study Baiocchi *et al.* found a significant correlation between mesenteric lymph node spread and the depth of colorectal wall invasion and the presence of metastases at the retroperitoneal lymph node stations [34]. Our experience underlines that whenever metastatic peritoneal spread in advanced ovarian cancer invades the colorectal wall, one can reasonably expect mesenteric lymph node involvement equal to or even greater than that in the typical pelvic and interaortocaval locoregional lymph node stations (Table 3). The incidence of these metastases correlates significantly with number of colorectal segments involved and the extent of colorectal resections undertaken (Table 3), as Lax *et al*. have underlined [28]. When we analyzed the site of the involved mesenteric lymph node stations, in 41.6% of the patients in whom cytoreduction required a rectal resection we found evident metastatic spread to the mesorectal lymph nodes, alone or in association with other sites. Our finding and the 39.4% recently reported by Gouy *et al.*[35], fully justify our decision to base our surgical strategy on the general criteria for surgical oncology. In patients in whom malignant disease infiltrated deep into the peritoneal pouch and intraperitoneal rectum, we used an approach analogous to that generally used for rectal cancer, namely an almost total mesorectal excision. In our series, neither the univariate nor the multivariate analysis identified overall lymph node status as a significant prognostic indicator. Although concurrent locoregional ovarian and mesenteric lymph node involvement worsened patients’ outcomes the difference failed to reach statistical significance. This finding agrees with O’Hanlan *et al*. and Lax *et al*. who reported that mesenteric lymph-node involvement worsened the outlook though not significantly [27,28]. Others more recently, Gallotta *et al*. [20], and Gouy *et al*. [35], reported similar survival in patients with and without spread to mesenteric lymph nodes. The observation that mesenteric lymph-node metastases especially if associated with locoregional lymph-node metastases worsen the prognosis, though not significantly, underlines the need for systematic lymphadenectomy, a procedure that is valuable for disease staging and is also therapeutically useful. If unrecognized lymph node disease were left *in situ*, patients with and without mesenteric lymph node spread would presumably no longer have a similar outcome.

Finally, if we analyze overall and disease-free survival in our series of patients with diffuse peritoneal spread from ovarian cancer in whom cytoreductive surgery included colorectal resection, our data rank high among the literature, especially given emerging evidence showing that diffuse ovarian peritoneal spread worsens outcomes [37].

## Conclusions

In patients with primary advanced or recurrent ovarian cancer with diffuse peritoneal metastases and colorectal involvement, the depth of colorectal wall invasion seems to be an independent prognostic factor equal to residual disease. Even though consensus now regards colorectal resections when needed as an accepted procedure during cytoreduction, the depth of colorectal wall invasion in these patients could be especially useful as a new criterion for stratifying patients into prognostic classes, a need that others have underlined [6]. When the diagnostic work-up discloses malignant disease invading the colorectal wall up to the mucosal layer, neoadjuvant chemotherapy could help to down-stage the disease.

A major concern that surgeons and pathologists should be aware of and deal with in patients with primary advanced or recurrent ovarian cancer involving the large bowel is dual locoregional and mesenteric lymphatic spread. Our experience corroborates previous evidence that the surgical technique for colorectal resection in these patients should strictly follow the criteria used for colorectal cancer [27,29].

## Competing interests

The authors declare they have no conflict of interest.

## Authors’ contribution

PS, MC, ADG participated in the conception and design of the study. SS, FA, DB, TC, AC, MLF analyzed and interpreted the data. PS, MC, ADG drafted and critically revised the manuscript. PS, MC, ADG, MLF gave final approval of the definitive version. All authors read and approved the final manuscript.
